# PrP turnover in vivo and the time to effect of prion disease therapeutics

**DOI:** 10.1101/2024.11.12.623215

**Published:** 2024-11-14

**Authors:** Taylor L Corridon, Jill O’Moore, Yuan Lian, Vanessa Laversenne, Briana Noble, Nikita G Kamath, Fiona E Serack, Abdul Basit Shaikh, Brian Erickson, Craig Braun, Kenney Lenz, Michael Howard, Nathan Chan, Andrew G Reidenbach, Deborah E Cabin, Sonia M Vallabh, Andrea Grindeland, Nina Oberbeck, Hien T Zhao, Eric Vallabh Minikel

**Affiliations:** 1 Program in Brain Health, Broad Institute of MIT and Harvard, Cambridge, MA, 02142, USA; 2 Weissman Hood Institute, Great Falls, MT, 59405, USA; 3 Ionis Pharmaceuticals, Carlsbad, CA, 92010, USA; 4 Charles River Laboratories, Worcester, MA, 01605, USA; 5 IQ Proteomics, Framingham, MA, 01702, USA; 6 Comparative Medicine, Broad Institute of MIT and Harvard, Cambridge, MA, 02142, USA; 7 McCance Center for Brain Health and Department of Neurology, Massachusetts General Hospital, Boston, MA, 02114, USA; 8 Department of Neurology, Harvard Medical School, Boston, MA, 02115, USA; 9 Prion Alliance, Cambridge, MA, 02139, USA; 10 Gate Bio, Brisbane, CA, 94005, USA

## Abstract

PrP lowering is effective against prion disease in animal models and is being tested clinically. Therapies in the current pipeline lower PrP production, leaving pre-existing PrP to be cleared according to its own half-life. We hypothesized that PrP’s half-life may be a rate-limiting factor for the time to effect of PrP-lowering drugs, and one reason why late treatment of prion-infected mice is not as effective as early treatment. Using isotopically labeled chow with targeted mass spectrometry, as well as antisense oligonucleotide treatment followed by timed PrP measurement, we estimate a half-life of 5–6 days for PrP in the brain. PrP turnover is not affected by over- or under-expression. Mouse PrP and human PrP have similar turnover rates measured in wild-type or humanized knock-in mice. CSF PrP appears to mirror brain PrP in real time in rats. PrP is more readily quantifiable in colon than in other peripheral organs, and appears to have a shorter half-life in colon than in brain. Our data may inform the design of both preclinical and clinical studies of PrP-lowering drugs.

## Introduction

Pharmacologic lowering of prion protein (PrP) delays onset and slows progression of prion disease in animal models^1–3^, consistent with PrP as the substrate for prion misfolding and the pivotal molecule in progression of this rapid neurodegenerative disease^4,5^. Inspired by this finding, a PrP-lowering antisense oligonucleotide (ASO) is now in a Phase I clinical trial (NCT06153966), with additional PrP-lowering modalities in preclinical development^6^.

Prion disease typically presents as a rapidly progressive dementia^7^, with the median patient in prior clinical trials surviving just ~2 months from randomization^8,9^, the time to effect of prion disease therapeutics could be a critical determinant of efficacy in the symptomatic population. In mouse models, PrP lowering is most effective when treatment is administered early in the silent incubation period (<78 days post-inoculation or dpi)^1^. Treatment after frank symptoms emerge (132 – 143 dpi) has extended survival primarily by increasing the time that animals are sick, without reverting any symptoms already accumulated^1,2^, and only a subset of late-treated animals benefit, while others succumb to disease on a similar timeframe as untreated animals^1^. One explanation is simply that PrP lowering cannot reverse existing neuronal loss. However, the observation that efficacy is limited even very late pre-symptomatic timepoints (105 – 120 dpi)^1^ led us to speculate that PrP turnover may be another factor limiting the efficacy of late treatment. ASOs target the PrP RNA for cleavage and degradation by RNase H1^1,10,11^, suppressing new PrP synthesis but leaving pre-existing PrP to be degraded according to its own half-life. PrP turns over rapidly in cultured cells^12,13^, but in vivo, reports are conflicting. A study using oral doxycycline to suppress expression of PrP under a Tet-off transgene determined the half-life of normally folded cellular PrP (PrP^C^) to be just 0.75 days in the brain^14^, while 2 mass spectrometry studies of mice fed isotopically labeled chow determined half-life estimates of 4.95 or 5.02 days in the brain^15,16^. Given that prion disease has heterogeneous subtypes with distinct rates of progression^7^, the difference between a half-life of <1 day versus 5 days would have a dramatic impact on the inclusion criteria needed to select for patients likely to have time to benefit from a PrP-lowering drug in clinical trials.

Here we set out to determine the half-life of PrP in brain, as well as to answer several related questions. Because some PrP-lowering drugs in development are expected to have systemic activity^6^, we sought to identify an organ or tissue that could be used as a proxy for peripheral target engagement in preclinical models, and further to determine the PrP half-life and therefore timeline on which target engagement can be observed in such a tissue. To mitigate translational risk due to amino acid sequence differences, depth of target suppression, or disease state, we sought to determine whether PrP half-life differs between human versus mouse PrP, in heterozygous knockout versus overexpressing animals, or in prion-infected versus naïve animals. Finally, because cerebrospinal fluid (CSF) is being used as a sampling compartment to reflect on brain PrP^5,17^, we also sought to determine the timeframe on which brain target engagement can be read out in CSF.

## Results

**Figure 1.**
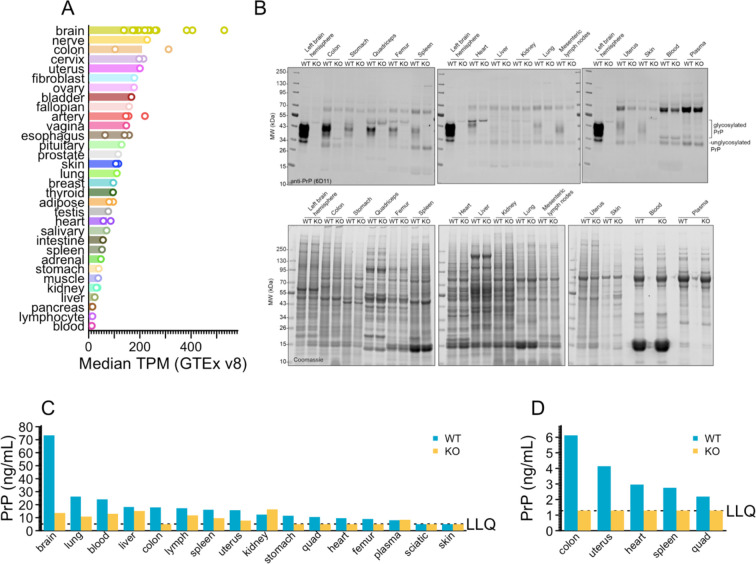
Nomination of colon as a tissue for peripheral PrP quantification. **A)** PRNP RNA expression in transcripts per million (TPM) in human tissues according to GTEx v8 public data. Each sub-tissue (e.g. brain – cerebellum) is represented by one point as the median TPM across all samples for that tissue, and each tissue (e.g. brain) is represented by one bar as the median of those medians. **B)** Western blot (top) and Coomassie (bottom) of organs all from the same 1 WT and 1 KO animal, 6D11 anti-PrP antibody, see Methods for details. **C)** Organs tested by PrP ELISA at a 1:100 final dilution (10% homogenates at a further 1:10). PrP ELISA as reported except using double the detection mAb concentration (0.5 μg/mL instead of 0.25 μg/mL). **D)** Organs tested by PrP ELISA at a 1:25 final dilution (10% homogenates at a further 1:2.5). PrP ELISA as reported except using double the detection mAb concentration (0.5 μg/mL instead of 0.25 μg/mL). See Figure S1 for further assay development.

We sought to identify a peripheral tissue in which we could quantify PrP. Analysis of human *PRNP* RNA expression data from Genotype-Tissue Expression project (GTEx v8)^18^ revealed that after brain and sciatic nerve, colon was the next tissue with the highest *PRNP* expression (Figure 1A). We dissected 15 organs from 1 wild-type and 1 PrP knockout mouse and analyzed them by Western blot. Brain exhibited far more PrP than any peripheral organ examined, but a strong band was identified centered at the expected molecular weight (~37 kDa) in colon, with weaker bands in stomach, quadriceps, heart, femur, spleen, and uterus, and faintly detectable bands in lung, lymph node, and skin. PrP was not detectable in liver, kidney, whole blood, or plasma (Figure 1B). Although homogenization efficiency and total protein loading varied between organs, Coomassie analysis revealed that the WT and KO animals were similarly loaded for any given organ (Figure 1C). When the same tissues were analyzed at a 1:100 wt/vol final dilution by our in-house ELISA^17^ with the EP1802Y/8H4 antibody pair, all tissues besides brain were near the lower limit of quantification (LLQ), with many reading above LLQ in the knockout animals, presumably due to matrix effects. Of any organ where the knockout tissue read out at LLQ, colon exhibited the strongest PrP signal in the wild-type animal (Figure 1D). Colon and 4 tissues with weaker signal were re-analyzed at a 1:25 wt/vol final dilution, yielding higher signal and confirming colon as the strongest tissue at 5-fold above LLQ (Figure 1E). Further assay development identified the best conditions for ELISA detection of colon PrP and established stability parameters for colon samples in this assay (Figure S1). These results led us to select colon as our proxy tissue.

**Figure 2.**
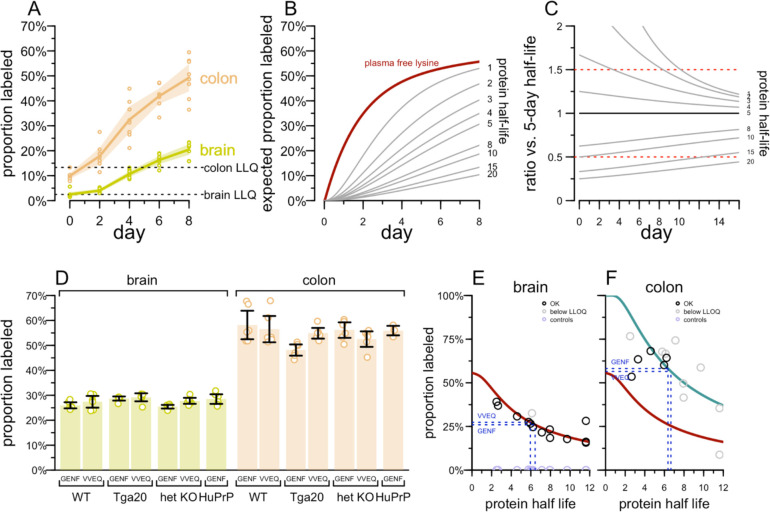
Determination of PrP half-life by targeted mass spectrometry. **A)** Accumulation of ^13^C_6_ label from chow in the VVEQ peptide in mouse brain and colon. **B)** The best fit to the proportion of plasma free lysine empirically found to be ^13^C_6_ labeled, as reported by Fornasiero (maroon), and the proportion of peptide expected to be labeled over time as a function of half-life (shown in days on the right side). See Methods > Labeled peptide accumulation models for details. **C)** The ratio of proportion labeled (B) for a peptide of each half-life compared to a peptide of 5-day half-life. Ratios of 0.5 and 1.5 are arbitrary landmarks highlighted to orient the eyes to a straight horizontal line. **D)** The proportion of PrP peptides VVEQ and GENF that are ^13^C_6_ labeled after 8 days as a function of mouse genotype. All differences are non-significant at Bonferroni-corrected P > 0.05, 2-sided T-test. **E)** Proportion labeled in brain (y axis) versus brain half-life previously reported by Fornasiero for all measured non-PrP peptides (circles); black = measured peptide signal above LLQ, gray = below LLQ. The maroon line represents the expected proportion labeled after 8 days as a function of half-life, based on the model from (B). The horizontal blue lines represent the proportion labeled observed for the two PrP peptides, and their vertical projection from the maroon curve down to the x axis represents the estimation of half-life from those proportion labeled measurements. **F)** As in (E), but for colon. The additional cyan curve represents the expected proportion labeled after 8 days if 100% of lysine available for protein synthesis is ^13^C_6_ labeled at all times.

We next sought to use targeted MS to measure turnover in both brain and colon. Initially we focused solely on the peptide VVEQMCVTQYQK (hereafter abbreviated VVEQ), the most readily quantified of any PrP tryptic peptide^19^. We fed wild-type mice with ^13^C_6_ lysine chow, sacrificed them at 0, 2, 4, 6, or 8 days — a range of timepoints around the hypothesized half-life of PrP based on prior mass spectrometry studies^15,16^ — measured VVEQ by targeted mass spectrometry, and quantified the percent labeled as the ratio of heavy peptide to heavy plus light. Due to lower overall PrP abundance (Figure S1), the LLQ in colon occurred at 13.3% labeling versus 2.5% for brain; nonetheless, heavy peptide was above LLQ in most samples by day 2, and in all samples on days 4–8 (Figure 2A). Heavy labeled peptide accumulated much more quickly in colon than in brain, with the two tissues reaching 49.3% and 20.5% respectively by day 8 (Figure 2A), potentially suggesting a shorter half-life in colon than in brain.

In order to interpret these data, we considered implications of the mathematical model for heavy label accumulation presented by Fornasiero et al^16^. Fornasiero measured the proportion of free lysine in mouse plasma that was labeled, and fit a model represented by the maroon line (Figure 2B). In this model, the proportion of lysine that is heavy labeled rises rapidly initially as dietary lysine becomes bioavailable, but then increases more slowly, reaching 55.7% by day 8, because the labeled dietary lysine is in competition with unlabeled lysine made available by catabolism of endogenous proteins. Because only a portion of free lysine is labeled, calculating the expected proportion of a peptide labeled as a function of its protein’s half-life revealed that according to this model, peptides from a protein with a 5-day half-life would be just 30.0% labeled by day 8, even though 62.1% of the protein would turn over in this time (Figure 2C).

With this in mind, we considered how many days of labeled chow consumption would best discriminate between shorter and longer half-lives. This analysis revealed a tradeoff: the theoretical difference between proportion labeled for a quick turnover protein and a slow turnover protein is maximized at early timepoints when the overall proportion labeled is still low enough that the precision of measurement near LLQ could be limiting. At later timepoints, the proportion labeled is higher, mitigating LLQ concerns, but the theoretical proportion labeled is less different. For discriminating half-lives near 5 days, an 8-day labeled chow experiment appeared to present a reasonable compromise between these tradeoffs.

To replicate and extend our results, we performed a multiplex targeted MS assay using VVEQ, another PrP peptide GENFTETDVK (hereafter abbreviated GENF), and a sampling of peptides from proteins whose brain half-lives as determined by Fornasiero^16^ ranged from 2.5 to 11.6 days, to serve as controls. Serial dilution of ^13^C_6_^15^N_2_ lysine synthetic peptides for this assay identified lower limits of quantification (LLQ) for each peptide; the mean heavy peptide area found in wild-type mice after 8 days of labeled chow was above LLQ for 17 peptides in brain and for 8 in colon, indicating the suitability of these peptides for this purpose (Figure S2).

We utilized multiple mouse lines (Table 1) to determine the impact of PrP amino acid sequence and expression level on half-life. For the multiplex MS assay, we fed wild-type, heterozygous PrP knockout, transgenic humanized (Tg25109; human PrP 129M), and transgenic overexpressing (Tga20 mouse PrP) mice with ^13^C_6_ lysine chow, sacrificed them at 8 days, and analyzed their brains by mass spectrometry. For either PrP peptide, measured in either tissue, the proportion labeled was not significantly different from wild-type for any genotype (Figure 2D).

When we plotted, for each peptide from various proteins, the proportion labeled in wild-type mouse brain at day 8 versus the half-life reported by Fornasiero (Figure 2E), we found excellent agreement with the theoretical proportion labeled expected based on the plasma free lysine curve (Figure 2C). Projecting the proportion labeled for the two PrP peptides (26.0% and 27.3% for GENF and VVEQ respectively) onto this curve (blue dashed lines) yielded estimates of 6.4 and 6.0 days respectively. Control mice fed unlabeled chow categorically had percent labeled at <0.5%, confirming specificity of the assay.

For colon, the proportion labeled at day 8 (58.2% and 56.5% for GENF and VVEQ respectively) was higher than even the proportion of plasma free lysine expected to be labeled at day 8 (55.7%), meaning that even if PrP turnover in colon were virtually instantaneous, the proportion labeled in colon could not be explained by the plasma free lysine model (Figure 2F). We considered the possibility that the colon absorbs lysine directly from the diet, bypassing the bloodstream. To test the extreme, we modeled the expected proportion labeled if 100% of lysine used for nascent protein synthesis is labeled from day 0 (cyan curve, Figure 2F). The proportion labeled for several peptides in colon aligned more closely with this curve than with the plasma lysine curve (maroon curve, Figure 2F), although data are limited because many of these brain-expressed proteins are below LLQ in colon. Under the extreme assumption that 100% of lysine were labeled from day 0, the PrP half-life inferred from this model would be 6.4 and 6.7 days for GENF and VVEQ respectively. These estimates are very close to the half-life estimates from brain, yet the assumption of 100% of lysine used for nascent protein synthesis being labeled instantly upon switching to heavy chow is implausible. If labeled lysine availability is higher in colon than in brain but still less than 100%, then our data would be consistent with somewhat more rapid turnover in colon than in brain, both for PrP and for a number of other brain-expressed proteins.

**Figure 3.**
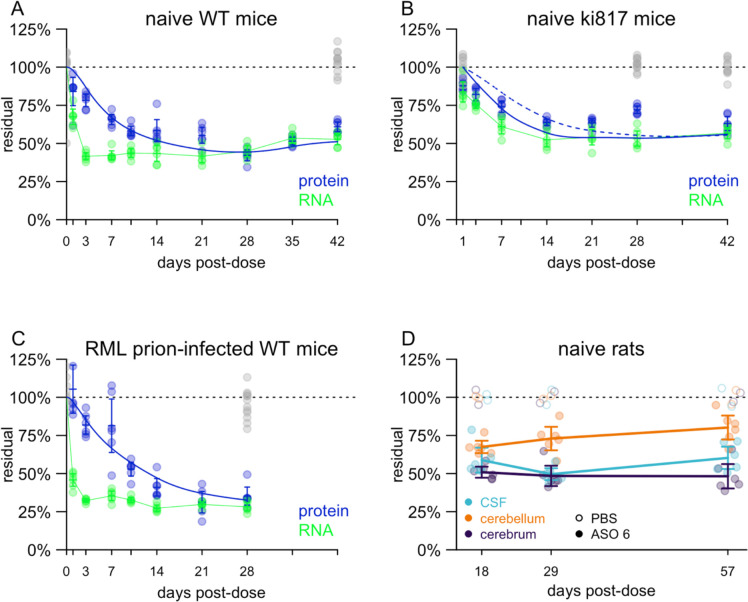
Determination of PrP half-life by ASO administration and timed sacrifice. **A)** Residual Prnp RNA and PrP protein (y axis), normalized to the mean of saline controls, at various timepoints (x axis) for wild-type mice after single ICV dose of 500 μg active ASO 6. Each point represents a whole brain hemisphere from one animal. All measurements in saline controls (both RNA and protein) are shown in gray. For each timepoint, line segments represent means and error bars represent 95% confidence intervals. The green curve represents linearly interpolated residual RNA concentration. The blue curve represents the exponential decay model fit to the data. **B)** As in (A) but for Ki817 human PrP 129V knock-in mice after a single dose 118 μg of ASO N. The solid blue line is the best fit model from the data in (B), while the dashed blue line represents a model using the half-life from (A) and the RNA data from (B). **C)** As in (A) but for wild-type mice infected with RML prions and treated with 300 μg ASO 6 at 105 dpi. **D)** Residual PrP in Sprague-Dawley rats treated with 1 mg of ASO 6 at day 0. Each point represents one animal, and for each timepoint, line segments represent means and error bars represent 95% confidence intervals. Long lines connect means of different timepoints.

We also sought to determine PrP’s half-life by an orthogonal method. We dosed naïve wild-type mice with 500 μg PrP-lowering active ASO 6^1^ (Table 2) by intracerebroventricular (ICV) injection at day 0 and then performed serial sacrifice to measure *Prnp* RNA and PrP protein in whole hemispheres at various timepoints post-dose (Figure 3A). Maximal RNA suppression was achieved within 3 days, while protein lagged, reaching its nadir at 28 days (Figure 3A). When we fit an exponential decay curve to the data, we obtained a half-life estimate of 4.8 days. Interestingly, while the model assumes a single rate of decay, the data do not fit such a paradigm perfectly. On one hand, we observed more knockdown at early timepoints than is explainable by a simple exponential decay model. For example, PrP protein was already down to 84% residual at day 1, when the model still predicts 98% residual. Conversely, we observed less pharmacodynamic activity at later timepoints than the model would predict: 58% residual at day 14, when the model predicts 52%. This could suggest that bulk PrP in whole brain hemisphere does not represent a single population with a uniform decay rate (see Discussion).

To confirm that there is no difference in half-life between mouse PrP and human PrP, we repeated the experiment in naïve humanized animals using a new human PrP (129V) knock-in mouse line termed Ki817 (Methods; Figure S4) treated with ASO N, which is potent against *PRNP* in both human and cynomolgus macaque^24^. Targeting 50% lowering to mirror the ASO 6 experiment above, we used a dose of 118 μg, which was the median effective dose (ED_50_) estimated in mouse cortex^25^. Due to a shortage of these humanized mice, we included fewer late timepoints in this experiment, thus inadvertently biasing the model towards the early timepoints where, in our previous experiment, knockdown was deeper than predicted by exponential decay. Perhaps as a result of this bias, the estimated half-life from these data was just 2.1 days (solid blue curve, Figure 3B). The half-life estimate of 4.8 from wild-type mice (dashed blue curve, Figure 3B) fit the data from later timepoints better.

To determine whether prion infection affects the half-life of PrP, we also performed the same experiment in RML prion-infected wild-type mice with a single ICV dose of 300 μg active ASO 6 at 105 dpi (Figure 3C). This yielded a similar picture as in naïve mice, with a half-life point estimate of 6.1 days. We note that we have not extensively tested the cross-reactivity of our PrP ELISA for PrP^Sc^; given the non-denaturing conditions of our ELISA, our assay is likely measuring primarily or exclusively PrP^C^.

To assess whether CSF PrP lags brain PrP, we used rats; the smaller CSF volume found in mice is challenging for robust PrP quantification^26^. After a single 1 mg ICV dose of active ASO 6 on day 0, rats were sacrificed at 18, 29, or 57 days post-dose and PrP was quantified in cerebrum (cortex and subcortex), cerebellum, and CSF (Figure 3D). Target engagement was deeper in cerebrum than in cerebellum at all timepoints, consistent with results from non-human primates and with the known difficulties in achieving strong ASO activity in cerebellar granule cells^24,27^. At all timepoints, the percent residual CSF PrP was in between that of cerebrum and of cerebellum, consistent with CSF reflecting some average of different brain regions. CSF PrP did not lag relative to cerebrum or cerebellum PrP, suggesting that it reflects brain PrP by 18 days post-dose, if not sooner.

## Discussion

Our data indicate that PrP’s half-life is between 4.8 to 6.4 days in brain parenchyma, regardless of PrP expression level, regardless of human or mouse PrP amino acid sequence, and regardless of prion infection status. Our estimate is in agreement with prior studies using mass spectrometry on the brains of mice fed isotopically labeled chow^15,16^. The conflicting report which determined a half-life of 0.75 days^14^ was performed in a transgenic Tet-off mouse line with PrP under a foreign promoter^28^. That mouse line apparently exhibited a PrP expression pattern different from endogenous PrP, as evidenced by the perinatal lethality observed when PrP was not suppressed with doxycycline treatment during pregnancy^28^. This might account for the different result. The mouse model also overexpresses PrP, and the animals in that study were infected with prions, but our data argue that neither of these factors is likely to explain the lower half-life estimate. In prion-infected mice dosed at 105 dpi and harvested from 106 to 133 dpi, we measured a half-life similar to that in uninfected mice, suggesting that the reported^29^ downregulation of normal PrP^C^ occurring by 120 dpi, if true, might not arise from accelerated turnover.

We find no evidence that CSF PrP lags brain PrP. In a previous study, where we analyzed only rat cerebrum (cortex and subcortex) we found that at 4 weeks post-dose, each 1% PrP lowering in CSF corresponded to 1.4% lowering in cerebrum^17^. Our present data, analyzing both cerebrum and cerebellum, indicate that this discrepancy might arise not from a lag in CSF PrP response to brain PrP concentration changes, but rather, from weaker target engagement in the cerebellum, which is also in contact with CSF.

We report that PrP in colon, while lower than brain, is quantifiable by ELISA. This is consistent with colon being the only tissue besides CNS and PNS to be affected in prion disease: patients with C-terminal truncating mutations that remove PrP’s GPI anchor and cause a gain of function through change in localization often experience chronic diarrhea misdiagnosed as inflammatory bowel disease for decades before the onset of peripheral neuropathy and then dementia^30,31^. Unlike those other peripheral tissues with lower PrP expression, colon is highly innervated; the relatively high PrP concentration there might reflect enteric nervous system expression. Colon PrP quantification could permit measurement of peripheral target engagement in animal studies of systemic PrP-lowering therapeutics. In mice fed isotopically labeled chow, heavy PrP peptide accumulates much faster in colon than in brain. This might indicate both more rapid turnover of PrP in colon, and higher availability of dietary labeled lysine in colon.

The diminution of PrP after ASO treatment is not perfectly modeled by a single exponential decay curve. Compared to the theoretical model, we observe deeper target engagement in the first few days after dosing than should be possible given that even the RNA has not been fully suppressed and less than one PrP protein half-life has passed; conversely, we observe less deep target engagement after 2–3 weeks than should be expected. One interpretation is that there are multiple populations of PrP that degrade according to different kinetics: for example, PrP might have different half-lives on different cell types, in different subcellular compartments, or in different multimeric states.

Our study has limitations. We do not have a perfect explanation for the higher rate of isotopic label incorporation in colon compared to brain. While our data suggest that it arises both from higher availability of dietary leucine and from a shorter half-life of PrP and possibly other proteins in colon, we have not empirically measured free ^13^C_6_ lysine in colon to confirm this. We also have not yet tested the kinetics of very deep PrP knockdown, below 50% residual. We lack a method for interrogating the half-life of PrP protein at the single cell level, so we do not know whether the rates may differ on distinct cell types. Most importantly, while we modeled human PrP in transgenic mice, we have not yet studied the half-life of PrP in humans.

Overall, our data confirm that PrP’s half-life is one rate-limiting step in the time to effect of therapeutics that act by inhibiting PrP synthesis. Ideally, drugs targeting PrP production could one day be combined with drugs increasing PrP catabolism or blocking its conversion to a misfolded form; pharmacologic proof-of-concept for such approaches in vivo is lacking but more research is merited. In the meantime, some sponsors might choose to enrich for patients with relatively high functional scores or relatively slower-progressing genotypes in order to see the strongest effect in trials. Accelerating diagnosis through better neurologist awareness, rapid referral, and shortened turnaround times for diagnostic tests will be critical for reaching patients early enough to achieve sufficient target engagement while they still possess quality of life.

## Methods

### Animals.

All animal experiments were approved by Institutional Animal Care and Use Committees (IACUC) at the Broad Institute (protocol 0162–05-17), Weissman Hood Institute (protocol 2024-AG-77) or Ionis Pharmaceuticals (protocol 2021–1176). All mice were of a pure C57BL/6N or mixed 6N/6J background. Details of ages and genotypes are provided in respective sections below. We utilized the ZH3 line of PrP knockout mice, the Tg25109 line of HuPrP 129M humanized mice, and the Tga20 line of PrP-overexpressing mice. Genotyping was performed by Transnetyx. Suggested primers for Tg25109 have been reported^22^. The Ki817 huPrP 129V mouse line is described here for the first time; see methods below and Figure S5. Tga20 mice were developed by Fischer et al^32^; the Tga20 transgene array was localized to the *Ptcra* gene locus by Taconic/Cergentis using targeted locus amplification (TLA)^33^; for details see Figure S6.

### Generation of human PrP knock-in mice.

Humanized KI *PRNP* mice (ki817) were generated by Taconic using ES cell targeting using CRISPR/Cas9. The targeting vector was constructed using human BAC RP11–61G12 for the human *PRNP* (129V) sequence and the mouse BAC RP23–369F12 for the homology arms. This targeting vector, a puromycin resistance cassette plasmid, and a plasmid containing Cas9 and guide RNAs against desired cut sites in the mouse genome were co-transfected into C57BL/6NTac embryonic stem cells. These were then selected, incorporated into embryos, and implanted to yield founders. The full targeting strategy is provided as a PDF in this study’s online data repository. The gRNA sequences used to target the mouse genomic region to be replaced with the human sequence were GGTCTGCTGATCCGACAACG and TAGAAGCTATGATGAACACC. The exact coordinates of human sequence incorporated into the mouse span from 306 bp upstream of the transcription start site to 1 bp downstream of the transcription end site (Figure S5).

### Isotopic labeling of mice.

Prior to study start, mice between ages of 21–23 weeks old were consolidated into cages based on genotype: wild-type, ZH3/+, Tga20 heterozygous, Tg25109 heterozygous on a ZH3/ZH3 background. One animal per genotype was set aside as a representative control. Mice were fed Mouse Express (unlabeled) irradiated mouse feed (Cambridge Isotope Laboratories, USA) for 7 days at libitum. On day 8, mice were switched to Mouse Express L-Lysine (^13^C_6_ , 99%) irradiated mouse feed (Cambridge Isotope Laboratories, USA) for 8 days at libitum. Animals had standard water access and no alternate food source was made available during the study. Amount of chow given each day was weighed prior to feeding and the remaining chow was weighed each day to estimate the amount of chow being eaten per animal per day. Animals were weighed on day 1 of unlabeled chow, day 1 of isotopically labeled chow and were harvested 24 hours after the last labeled chow refresh.

### Organ harvest.

Animals were weighed directly before harvesting. Animals were euthanized via CO_2_ asphyxiation and disarticulation of the skull and cervical vertebrae was utilized as a secondary measure. Left and right brain hemispheres were collected by hemi-secting the full intact brain with a scalpel on ice, hemispheres were collected into separate tubes and flash frozen on dry ice. Left and right sciatic nerves were collected in length from the proximal hip to distal knee and flash frozen on dry ice. Colon sections were harvested in length from the caudal end of the ascending colon to the middle of the transverse colon and flash frozen on dry ice. All samples were stored at −80C and sent for LC-MS in the form of frozen, intact tissue.

### Immunoblots.

All samples were homogenized in 0.2% CHAPS. All organs were homogenized at 10% wt/vol, except for sciatic nerve, which was 5% wt/vol due to limited sample mass, and blood and plasma, which were not homogenized. Each sample was then diluted 4-fold into RIPA buffer with protease inhibitors, vortexed for 1 minute, centrifuged 14,000 × G for 10 minutes at 4°C and then supernatants were diluted a further 4-fold into 4X LDS+TCEP and incubated 5 minutes at 95°C. 10 μL of this solution was then loaded per lane and run on a SDS-PAGE gel (4–12% Bis-tris NuPAGE No. NP0323BOX) in MES buffer for 40 minutes at 180V. Proteins were either Coomassie stained or transferred to iBlot using 20V for 7 minutes, cooled for 3–5 minutes, blocked with TBS blocking buffer (Licor No. 927–60001) for 1 hour at room temperature. 6D11 anti-PrP primary antibody (Biolegend no. 808003) was diluted 1:1000 in 0.2% Tween and incubated overnight at 4°C. Blots were then rinsed 4x with TBST, and goat anti-mouse IRDye 800CW (Licor No. 926–32210) was diluted 1:10,000 in TBS with 0.2% Tween, incbuated 1 hour at room temperature with rocking, and washed 4x with TBST. Blots were imaged at 800 and 700 nm on a Licor Odyssey CLx Infrared Imaging System.

### Tissue homogenization for ELISA.

Each brain hemisphere was added to a 7 mL Precellys tube with pre-loaded zirconium oxide beads (Precellys, Bertin, USA) and homogenized in ice cold 0.02% CHAPS in 1x PBS with protease inhibitors (1 Roche cOmplete tablet 4693159001, Millipore Sigma, USA, per 10 mL of buffer) using 3× 40 seconds pulses at 6,000 rpm in the Bertin Technologies Precellys Evolution Touch Homogenizer (Bertin, USA). The final protocol for colon homogenization (after optimization, see Figure S1) used 2 mL Precellys tubes and 5× 40 second pulses at 8,000 rpm. Homogenates were aliquoted into multiple 40 μL aliquots for protein analysis and 1 mL aliquots as a backup stock, flash frozen on dry ice and stored at −80°C until further analysis.

### PrP ELISA.

PrP concentration in the rat and murine brain hemispheres was measured using a previously published PrP ELISA^17^. The capture antibody, EP1802Y (ab52604, Abcam, USA), is incubated in a clear 96-well plate overnight at 4C. After blocking and sample incubation, biotinylated 8H4 antibody (ab61409, Abcam, USA) is used for detection with streptavidin-HRP (Pierce High Sensitivity, 21130, Thermo Fisher Scientific, USA) and TMB substrate (7004P4, Cell Signaling Technology, USA). Brain homogenates and QCs were diluted to 1:200 final concentration for the assay; the final protocol for colon homogenization (after optimization in Figure S1) utilizes a 1:100 final dilution. Recombinant mouse PrP, (MoPrP23–231) prepared as described^34,35^ was used for the standard curve. Average residual PrP was calculated by dividing the amount of residual PrP in each treated brain by the mean concentration of residual PrP in the vehicle and/or no dose control brains from the same study and time point. For the colon data in Figure 1D–E, the detection mAb concentration was doubled (0.50 μg/mL instead of 0.25 μg/mL), but with further assay development (Figure S1) we were able to revert to the original 0.25 μg/mL concentration and maintain the ~5-fold margin above LLQ.

### Prnp qPCR.

The qPCR procedure has been described previously^1^. *Prnp* RNA levels were normalized first to housekeeping gene *Ppia* then to the mean of PBS-treated controls. Primers are as follows. Prnp forward: TCAGTCATCATGGCGAACCTT, reverse: AGGCCGACATCAGTCCACAT, probe: CTACTGGCTGCTGGCCCTCTTTGTGACX. Ppia forward: TCGCCGCTTGCTGCA, reverse: ATCGGCCGTGATGTCGA, probe: CCATGGTCAACCCCACCGTGTTCX.

### Mouse inoculation.

Mouse inoculation has been described previously^1^. Animals were freehand inoculated halfway between the right ear and right eye, approximately 1 mm right of the midline, with 30 μL of a 1% (wt/vol) brain homogenate from terminally sick RML prion-infected mice.

### Mouse intracerebroventricular (ICV) injection.

Mouse ICV was similar to that described previously^1^. ASOs were diluted to 500 μg in a 10 μL dose volume in dPBS (Gibco 14190) and administered into CSF by bolus ICV injection in stereotaxis (ASI Instruments, SAS-4100). Positioning utilized 18° ear bars in ear canals and incisors in the mouse adapter tooth bar, adjusted to −8 mm. A 1 cm incision was made and the periosteum was scrubbed with sterile cotton-tipped applicators in order to reveal bregma. Drug was administered in Hamilton syringes (VWR 60376–172) fitted with 22-gauge Huber needles (VWR 82010–236). The needle was aligned to bregma and then moved 0.3 mm anterior, 1.0 mm right, and then downward either 3.0 mm past where the bevel disappeared into the skull or 3.1 mm past where the tip of the needle first touched the skull. Liquid was ejected over 10 seconds and the needle withdrawn 3 minutes later while applying downward pressure on the skull with a cotton-tipped applicator. Incisions were closed with a horizontal mattress stitch (Ethicon 661H suture). Animals recovered from the anesthesia in their home cages on a warming pad.

### Rat ICV injection.

The rat ICV injection was as described previously^17^. The procedure is similar to that for mice (see above) except that it utilizes 27° atraumatic ear bars (ASI Instruments, EB-927), with coordinates: riser −6 mm, 1 mm caudal, 1.5 mm right, 3.7 mm from the surface of the brain into the lateral ventricle. A bore hole was first drilled using a sterile 1 mm × 33 mm drill bit (McMaster Carr, 5058N51) in a hanging-style handpiece (McMaster Carr, 4454A14) held in a stereotactic handpiece holder (ASI Instruments, DH-1000). Injection volume was 30 μL in a gastight 1710 small RN syringe (Hamilton 81030). Incision closure utilized 5-O monofilament suture (Ethilon 661G-RL).

### Targeted mass spectrometry.

Peptides terminating in lysine were nominated based on prior mass spectrometry work^19^. Single peptide quantification for VVEQMCVTQYQK (Figure 2A) was performed at Charles River Labs (Worcester, MA). Serial dilution experiments determined an LLQ of 0.111 ng/mL (ng of peptide per mL of homogenate), corresponding to 30.6 fmol/mg (fmol of peptide per mg of total protein). Multiplex quantification of VVEQMCVTQYQK, GENFTETDVK, and the control peptides from other proteins (Figure 2D–F) was performed at IQ Proteomics (Framingham, MA). Details of the LCMS methods are provided in the supplementary material.

### Labeled peptide accumulation models.

Free lysine in plasma was assumed to follow the fit described by Fornasiero^16^: H = 1–0.503*(exp(−t*ln(2)*0.799))-0.503*exp(−t*ln(2)/39.423), where H means the proportion of free lysine that is ^13^C_6_ labeled, and exp(x) signifies e^x^. Using this formula we calculated the percent labeled at every timepoint from 0 to 16 days using increments of dt = 0.01 days. We then calculated the accumulation of ^13^C_6_ lysine label in peptides using numerical integration as follows. For parameter lambda (λ), defined as λ = ln(2)/t_1/2_ the turnover of protein in an arbitrarily small unit of time dt is λ*dt. For a 5-day half-life protein, for example, λ = ln(2)/5 = 0.14, so that every 0.01 days, 0.01*ln(2)/5 = 0.0014 (expressed as a proportion) or 0.14% of protein is catabolized, and 0.14% of the original amount is produced to replace it. The protein begins 100% unlabeled, and label accumulates as unlabeled protein is catabolized and a greater and greater proportion of the nascent protein is ^13^C_6_ labeled. The uniroot function in R was used to perform the inverse operation — estimating a half-life from the observed proportion labeled at a given timepoint.

### Exponential decay model.

All residual *Prnp/PRNP* RNA and PrP protein measurements were normalized, respectively, to the mean RNA and protein measurements in the untreated animals across all timepoints so that all measurements are on a scale from 0% (complete knockdown) to 100% (normal levels). Observed *Prnp/PRNP* RNA measurements in brain were linearly interpolated using the approx function in R to yield point estimates of residual RNA at units of dt = 0.01 days. Similar to the approach described above, the residual protein (P) at any given timepoint was computed numerically as a function of RNA (R) and the exponential decay parameter λ = ln(2)/t_1/2_ value. Catabolism of protein is proportional to the amount of protein in the previous time increment, while synthesis of protein is proportional to RNA in the previous time increment. So, at any time t, the protein catabolized is P_t-1_ * λ * dt, and the protein synthesized is R_t-1_ * λ * dt. Thus, the change in protein dP = R_t-1_ * λ * dt - P_t-1_ * λ * dt. The amount of protein at time t is P_t_ = P_t-1_ + dP. With this function in hand, another function was written to calculate the residuals of the actual data as compared to this model. Then, the <Monospace>nls.lm</Monospace> function in R was used to determine the λ value that minimizes those residuals, this fitting the model. A time increment of dt=0.01 and a starting guess of λ = 0.14, corresponding to a half-life of 5 days, were used in fitting the model.

### Statistics, source code and data availability.

All analyses were conducted using custom scripts in R 4.4.1. Exponential decay and label accumulation models are described above. Differences between genotypes were compared using a 2-sided T test and Bonferroni corrected for 10 tests. All error bars or shaded areas shown are 95% confidence intervals. P values of < 0.05 were considered significant. Raw data and source code sufficient to reproduce all figures and statistics in this manuscript will be made available at github.com/ericminikel/halflife.

## Supplementary Material

Supplement 1

Supplement 2

## Figures and Tables

**Table 1.** Mouse lines used in this study.

Name	PrP amino acid sequence	Expression level (fold wild-type)	Genomic location	Reference
ZH3/+	MoPrP-A†	0.5x	*Prnp*	^21^
Tg25109*	HuPrP 129M	1.1x	*Frdm6/Tmx1*	^22^
Ki817	HuPrP 129V	1.0x	*Prnp*	This study Fig S5
Tga20*	MoPrP-A	2.4x	*Ptcra*	^23^, this study Fig S6

* Maintained on a background of homozygous ZH3/ZH3 PrP knockout.

† MoPrP-A refers to the mouse reference genome PrP sequence as found in C57BL/6N and most other commonly used mouse strains (as opposed to the MoPrP-B allele, containing the two substitutions L108F and V189T, found in certain strains^20^).

**Table 2.** Antisense oligonucleotides used in this study.

ASO	sequence and chemistry	target	ref
ASO 6	mCToTomCoTATTTAATGTmCAoGoTmCT	mouse/rat *Prnp* 3’ UTR	^11^
ASO N	GTomCoAoToAoATTTTmCTTAGmCoTAmC	human/NHP *PRNP* intron	^24^

Black: unmodified DNA (2’H). Orange: 2’ methoxyethyl (2’MOE). Blue: 2–4’ constrained ethyl (cEt). Unmarked backbone linkages: phosphorothioate (PS). Linkages marked with o = phosphodiester (PO). mC: 5-methylcytosine.
